# Transtibial limb loss does not increase metabolic cost in three-dimensional computer simulations of human walking

**DOI:** 10.7717/peerj.11960

**Published:** 2021-08-04

**Authors:** Ross H. Miller, Elizabeth Russell Esposito

**Affiliations:** 1 Department of Kinesiology, University of Maryland, College Park, MD, United States of America; 2 Neuroscience and Cognitive Science Program, University of Maryland, College Park, MD, United States of America; 3 Extremity Trauma and Amputation Center of Excellence, Fort Sam Houston, TX, United States of America; 4 Center for Limb Loss and Mobility, Seattle, WA, United States of America; 5 Department of Mechanical Engineering, University of Washington, Seattle, WA, United States of America

**Keywords:** Limb loss, Prosthesis, Military, Muscle strength, Metabolic cost, Computer simulation, Direct collocation, Optimal control

## Abstract

Loss of a lower limb below the knee, *i.e.*, transtibial limb loss, and subsequently walking with a prosthesis, is generally thought to increase the metabolic cost of walking *vs*. able-bodied controls. However, high-functioning individuals with limb loss such as military service members often walk with the same metabolic cost as controls. Here we used a 3-D computer model and optimal control simulation approach to test the hypothesis that transtibial limb loss in and of itself causes an increase in metabolic cost of walking. We first generated *N* = 36 simulations of walking at 1.45 m/s using a “pre-limb loss” model, with two intact biological legs, that minimized deviations from able-bodied experimental walking mechanics with minimum muscular effort. We then repeated these simulations using a “post-limb loss” model, with the right leg’s ankle muscles and joints replaced with a simple model of a passive transtibial prosthesis. No other changes were made to the post-limb loss model’s remaining muscles or musculoskeletal parameters compared to the pre-limb loss case. Post-limb loss, the gait deviations on average increased by only 0.17 standard deviations from the experimental means, and metabolic cost did not increase (3.58 ± 0.10 J/m/kg pre-limb loss vs. 3.59 ± 0.12 J/m/kg post-limb loss, *p* = 0.65). The results suggest that transtibial limb loss does not directly lead to an increase in metabolic cost, even when deviations from able-bodied gait mechanics are minimized. High metabolic costs observed in individuals with transtibial limb loss may be due to secondary changes in strength or general fitness after limb loss, modifiable prosthesis issues, or to prioritization of factors that affect locomotor control other than gait deviations and muscular effort.

## Introduction

Numerous studies report on gait deviations and a high metabolic cost of walking in individuals with lower limb amputation (*e.g.*, Pinzur et al., 1992; Sanderson & Martin, 1997; Rábago & Wilken, 2016). These deviations from the mechanics and energetics of able-bodied gait are suspected to affect mobility, quality of life, and risk for chronic musculoskeletal conditions after limb loss (Molen, 1973; Gailey et al., 2008). Minimizing gait deviations and maintaining a reasonably economical gait are therefore important goals of rehabilitation and prosthesis prescription after limb loss. Deviations from able-bodied gait mechanics and energetics are often presumed to result directly from the amputation. However, the studies documenting these deviations are necessarily cross-sectional in nature. There are no longitudinal data on actual biomechanical and metabolic changes pre-to-post limb loss.

Longitudinal studies on gait mechanics pre- *vs.* post-limb loss are impractical but can be approximated in computer simulations (Zmitrewicz, Neptune & Sasaki, 2007; Handford & Srinivasan, 2016). In a previous study, motivated by observations that high-functioning children and military service members with transtibial limb loss do not have a greater metabolic cost of walking than able-bodied controls (Jeans, Browne & Karol, 2011; Russell Esposito et al., 2014; Jarvis et al., 2017), we used optimal control simulations to perform an *in silico* longitudinal study of walking mechanics and energetics pre- *vs.* post-limb loss. The results suggested that maintenance of residual limb muscle strength can maintain the pre-limb loss metabolic cost of walking, with only small deviations from the pre-limb loss gait mechanics (Russell Esposito & Miller, 2018). However, the computer model in this study was limited to only the sagittal plane. Many of the reported gait deviations in individuals with limb loss occur outside the sagittal plane (Rábago & Wilken, 2016), and lateral balance in normal human walking appears to have a modest metabolic cost (Donelan et al., 2004). Neglecting three-dimensional motion when making inferences on the energetics of walking with a prosthesis may therefore be a questionable assumption.

Therefore, the purpose of this study was to perform an *in silico* longitudinal study pre- and post-transtibial limb loss using a three-dimensional musculoskeletal model to determine the change in metabolic cost, with its typical definition as the energy expended to translate a unit mass by a unit distance (Schmidt-Nelson, 1972; Tucker, 1975). Based on previous experiments and simulations (Russell Esposito et al., 2014; Russell Esposito & Miller, 2018), we hypothesized that the metabolic cost of walking with a transtibial prosthesis post-limb loss would be similar to the pre-limb loss metabolic cost when the pre-limb loss muscular properties and body composition were maintained.

## Methods

### Pre-limb loss model

**Figure 1:**
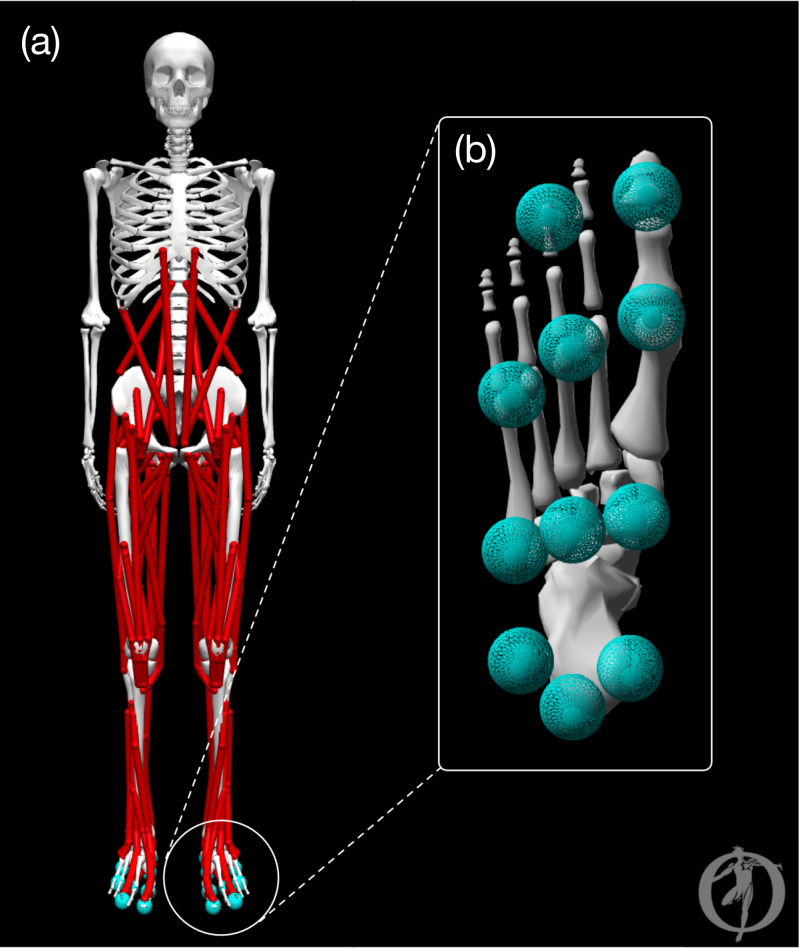
Graphic of the OpenSim model. The pre-limb loss model visualized in OpenSim software. (A) Full body with all joint angles set to zero. (B) Plantar surface of the foot with contact spheres.

The model was a modified version of the OpenSim (Delp et al., 2007) model described by Rajagopal et al. (2016). The pre-limb loss model is visualized in Fig. 1 and consisted of pelvis, trunk, thigh, shank, foot, toes, upper arm, and forearm rigid bodies that articulated with 31 degrees of freedom. The lumbar joint and lower limb joints were actuated by 84 total muscles (42 bilateral pairs). The shoulder and elbow joints were actuated by ideal torque generators with activation dynamics. The model had a total body mass of 75.4 kg and height 1.75 m. Several changes or additions were made to the original model, which lacked some elements necessary or beneficial for optimal control simulations, to make it well suited for direct collocation simulations in Moco software (Dembia et al., 2020):

1. The original “Millard2012Equilibrium”-type muscles were replaced with “DeGrooteFregly2016”-type muscles, which have activation and contractile dynamics suitable for gradient-based optimization in Moco (De Groote et al., 2016).
2. Wrapping objects that defined muscle paths on the skeleton were replaced with “Conditional Path Points” that gave similar relationships between the skeletal pose and muscle lengths and moment arms. Moco does not currently support wrapping objects.
3. The studies that motivated the present study examined samples of young men (Russell Esposito et al., 2014; Jarvis et al., 2017). Therefore, muscle optimal fiber lengths and unloaded tendon lengths were adjusted so that the model produced maximum isometric joint torques at average joint angles for young men from Anderson, Madigan & Nussbaum (2007). Specifically, optimal fiber length was adjusted unless the adjustment required shortening it to a length that prevented the muscle from producing force across a realistic range of motion, in which case unloaded tendon length was adjusted instead. The adjustments made from the original model’s parameters were small, *e.g.*, under three cm for the longest muscles in the model.
4. The original model did not include muscle-specific time constants for activation dynamics. These muscle activation dynamics time constants were defined as functions of muscle mass and fast-twitch fiber ratio using fiber-type data from Miller (2018) and time constant functions from Winters & Stark (1985).
5. The original model did not include passive forces limiting joint ranges of motion beyond the parallel elastic components of muscles. Therefore, coordinate forces in the form of passive torsional spring-dampers were added to the joints representing lumped effects of ligaments and other non-muscular joint structures on range of motion (Anderson, 1999).

Additionally, ground contact was modeled by 11 sphere-shaped Hunt-Crossley contact elements (Sherman, Seth & Delp, 2011) located on the plantar surface of each foot (Fig. 1). The modulus of the contact elements (3.06 MPa) and damping coefficient (2.0 s/m) were set so that the heel contact sphere deformation and energy return were similar to the heel region of a human foot in an athletic shoe (Aerts & De Clercq, 1993). The frictional contact forces were a Stribeck friction model (Sherman, Seth & Delp, 2011) with static, dynamic, and viscous friction coefficients of 0.8, 0.8, and 0.5, respectively, and transition velocity 0.2 m/s.

**Figure 2:**
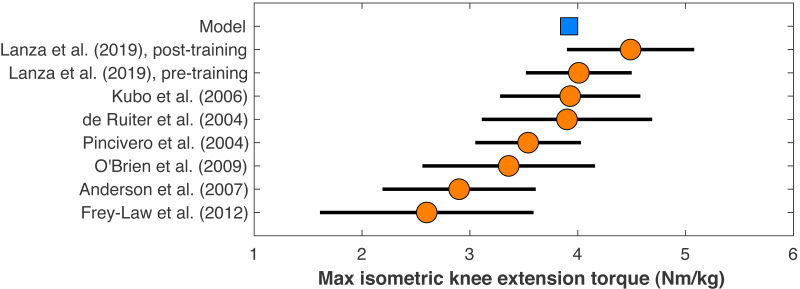
Maximum isometric knee extension torque of the model. The model’s maximum isometric knee extension torque when simulating typical conditions from human dynamometry tests for hip posi- tion, agonist activation, and antagonist activation (Kubo et al., 2004). Circles are means with standard de- viation bars for similar dynamometry experiments on healthy young men. Data from Lanza, Balshaw & Folland (2019) are before and after four weeks of quadriceps strength training. Other data are from De Ruiter et al. (2004), Frey-Law et al. (2012), O’Brien et al. (2009), and Pincivero et al. (2004).

The model’s muscle volumes were determined as functions of the model’s mass and height using average MRI measurements of muscle volume for healthy young adults from (Handsfield et al., 2014). Maximum isometric forces were the determined by dividing the volume by the muscle’s optimal fiber length to determine the physiological cross-sectional area, and multiplying this area by a specific tension of 60 N/cm^2^, the same value assumed in the original model (Rajagopal et al., 2016). This specific tension is larger than the values typically determined from *in vitro* single-fiber experiments, which average ∼13 ± 6 N/cm^2^ in human muscle (Rospars & Meyer-Vernet, 2016), but is similar to the *in vivo* result on human quadriceps of 55 ± 11 N/cm^2^ for healthy adult men from O’Brien et al. (2010). The model’s maximum isometric knee extension torque when emulating typical conditions of human dynamometry experiments on hip position, voluntary activation, and antagonism (Kubo et al., 2006), was 3.92 Nm/kg, which compares reasonably well to dynamometry experiments on human subjects that reported torque–angle data (Fig. 2).

Muscle metabolic rates were calculated as functions of muscle activation and fiber velocity using the model described by Umberger, Gerritsen & Martin (2003). The muscle rates were summed and added to an assumed basal metabolic rate of 1.0 W/kg to determine the model’s whole-body metabolic rate. The metabolic rate was then averaged over time and divided by average walking speed to determine the metabolic cost, *i.e.*, the energy expended per unit distance traveled.

### Post-limb loss model

The model of limb loss and a generic prosthesis was based on our previous 2-D simulations (Russell Esposito & Miller, 2018). The 13 muscles spanning the right ankle were removed and the double-exponential joint torque–angle relationships representing the biological ankle, subtalar, and toe joints were replaced by linear relationships of the form:

(1) \begin{eqnarray*}\tau =-k\theta -b\dot {\theta }\end{eqnarray*}

where *τ* is the passive moment at the joint, *θ* is the joint angular position, and *k* and *b* are the stiffness and damping constants. The mass and moment of inertia of the prosthetic leg’s bodies below the knee were reduced to 65% and 40%, respectively, of the intact leg values, representing the typical mass and mass distribution in a transtibial prosthesis (Smith & Martin, 2013). To compute the post-limb loss model’s basal metabolic rate, the minimum heat rates of all 84 pre-limb loss muscles (1.0 W per kg muscle mass; (Umberger, Gerritsen & Martin, 2003) were subtracted from the total basal metabolic rate (1.0 W per kg biological mass) to determine the non-muscular basal metabolic rate (54 W average). The non-muscular rate was assumed to be unchanged post-limb loss, and the minimum heat rates of the remaining 71 post-limb muscles were added to the non-muscular rate to determine the post-limb loss basal metabolic rate. The post-limb loss model was otherwise identical to the pre-limb loss model.

A limitation of this prosthesis model is that the prosthesis was rigidly attached to the residual limb, which is more similar to an osseointegrated prosthesis than a traditional prosthesis with deformation at the socket-residuum interface. Currently, osseointegration is primarily used in patients with transfemoral limb loss who have negative outcomes using a traditional prosthesis, although transtibial osseointegration is feasible (Frossard, Leech & Pitkin, 2020). In model development, a model that allowed pistoning between the prosthesis and residual limb was tested. The force–deformation relationship of the distal residuum was a double-exponential:

(2) \begin{eqnarray*}F=-{a}_{1}\exp \nolimits \left( {a}_{2}y \right) +{a}_{1}\exp \nolimits \left( -2{a}_{2}y \right) -b\dot {y}\end{eqnarray*}

where *F* is the soft tissue load at the socket-residuum interface and *y* is the soft tissue deformation along the long axis of the residuum. Values for *a*_1_ = 7.8 and *a*_2_ = 350 were set to approximate the average deformation profile from 0–100% bodyweight loading from fluoroscopy data (Darter, Sinitski & Wilken, 2016). Damping *b* = 100 N/(m/s) was set to suppress high-frequency oscillations of the prosthesis during the swing phase. Adding pistoning changed the model’s metabolic cost of walking by only 0.5% and did not affect its accuracy in tracking experimental gait data, so the rigid socket-residuum interface without pistoning was used for simplicity.

### Simulations

#### Subjects

As a computer simulation study, the “subjects” in the present study were not specific human participants but were instances of the model described in ‘Pre-Limb Loss Model’–‘Post-Limb Loss Model’ with different parameter values. “Subject #1” used the default values of the pre-limb loss model’s parameters. To generate additional pre-limb loss subjects, a scaling factor between 0.75–1.25 was drawn randomly from a standard normal distribution and scaled all the model’s maximum isometric muscle forces to adjust the model’s overall strength, *e.g.*, if the scaling factor was 1.12, every maximum isometric force was multiplied by 1.12. Smaller random scaling factors of 0.95–1.05 that varied for each individual parameter were then applied to the scaled maximum isometric muscle forces, tendon slack lengths, and body segment masses. The body segment masses were constrained to still sum to the model’s original mass and all parameter values were still required to be bilaterally symmetric, as in the original model. The ranges over which these parameters were set to produce a range of pre-limb loss metabolic costs of roughly 3.4–3.8 J/m/kg. These models were then converted to post-limb loss models (‘Post-Limb Loss Model’). For each subject’s post-limb loss model, a random fraction of 20–80% of the prosthetic limb’s shank mass was assumed to be biological mass to represent a range of residual limb sizes.

The muscle maximum isometric forces and tendon slack lengths were perturbed because Hill model output is very sensitive to these parameter values (*e.g.*, Scovil & Ronsky, 2006). The segment mass distributions were perturbed because confidence in their values for a human subjects experiment is typically low. Other unmentioned parameters that could feasibly affect metabolic cost were not perturbed. The element of “subjects” in this study should essentially be viewed as a limited sensitivity analysis, hopefully providing some confidence that the results are due to the independent variable (limb loss) and not to the values assumed for parameters that are difficult to assign on a subject-specific basis with confidence. Similarly, the “subjects” here are perhaps better viewed as different parameter sets that may feasibly be assigned to an individual, or as different possible baseline fitness levels of an individual, rather than distinct individuals.

The minimum detectable effect of interest was a 2% change in metabolic cost, which is the minimum detectable change reported in testing of a modern portable pulmonary gas exchange unit due to measurement error alone, in the absence of any human biological variability between tests (Guidetti et al., 2018). The motivation for this choice of 2% is that smaller changes would be difficult to measure on human participants even if they were real changes. Simulation results from 20 subjects suggested at least 26 subjects were needed to detect the 2% effect as significant with error rates of 5% for both Type I and Type II errors. A total of 36 subjects were therefore used. In the present data, 2% equaled an effect size of 0.71, or ±0.068 J/m/kg in raw units of metabolic cost.

#### Overview

The models described above were used to simulate periodic strides of walking with stride duration *T* = 1.0 s and speed 1.45 m/s, the averages from healthy young adults measured by Miller et al. (2014). The simulations were posed as optimal control problems, finding the time-varying muscle excitations and associated model states that minimized a cost function *J* that was the weighted sum of gait deviations plus muscular effort:

(3) \begin{eqnarray*}J= \frac{1}{T} \int \nolimits \nolimits _{0}^{T} \left[ \frac{1}{{N}_{1}} \sum _{i=1}^{{N}_{1}}{ \left( \frac{{x}_{i} \left( t \right) -{\mu }_{i} \left( t \right) }{{\sigma }_{i}} \right) }^{2}+ \frac{w}{{N}_{2}\Delta x} \sum _{i=1}^{{N}_{2}}{m}_{i}{u}_{i}^{2} \left( t \right) \right] dt.\end{eqnarray*}

The first term on the right-hand side of Eq. (3) is the “gait deviations”: ${x}_{i} \left( t \right) $ is the value of model variable *i* at time *t*, ${\mu }_{i} \left( t \right) $ is the mean of the analogous variable from the experimental gait analysis data of Miller et al. (2014), and *σ*_*i*_ is the standard deviation between-subjects of the experimental data, averaged over the stride cycle. The *N*_1_ = 37 variables included in this term were the six translations and rotations of the pelvis, the 25 joint angles, and the 3-D components of each ground reaction force (GRF). The square root of this term after dividing the integral by *T* gives the average tracking error, in multiples of SD. For example, a tracking error of 1.0 indicates the model’s gait mechanics were 1.0 SD away from the experimental means when averaged over all timesteps and all tracking targets. Hip internal rotation, subtalar motion, and non-sagittal shoulder motions did not track experimental data due to low confidence in accurately measuring these motions with skin marker-based motion capture. These motions tracked target angles of 0° to minimize their ranges of motion, which are typically small in walking. Assigning no tracking targets for these degrees of freedom tended to produce unusual movements to track other targets unnecessarily well, *e.g.*, the GRF. The hands were constrained to not pass through the pelvis. Lumbar joint motions were also not tracked. Instead, deviations of the trunk angle from the global axes were minimized. The standard deviations of the experimental data were still used for *σ*_*i*_ as they were presumed to reflect the variability of the underlying motions.

The second term on the right-hand of Eq. (3) is the “muscular effort per unit distance”: ${u}_{i} \left( t \right) $ ranges from 0-1 and is the excitation of actuator *i* at time *t*, *m*_*i*_ is the mass of muscle *i*, and Δ*x* is the horizontal displacement of the model during the stride. *N*_2_ = 92 is the number of actuators in the model: 84 muscles and eight torque generators. This “muscular effort” term is a surrogate of metabolic cost and was used in the cost function instead of metabolic cost itself because (i) this version of the Moco software could not include metabolic cost in the cost function, and (ii) it is unclear if the mechanics of normal walking are consistent with a control policy that minimizes metabolic cost itself (Bertram, 2005; Ackermann & Van den Bogert, 2010). Smaller gait deviations will generally require greater muscular effort, assuming the model’s minimum-effort gait is not identical to the tracking targets. The weighting constant *w* determines the emphasis of the optimization on minimizing effort *vs.* deviations. Values of *w* = 50–200$/\bar {m}$, where $\bar {m}$ is the average muscle mass, produced realistic metabolic costs in the range of 3.0–3.8 J/m/kg, typical of healthy young adults at comfortable walking speeds (Das Gupta, Bobbert & Kistemaker, 2019), with average tracking errors typically under 1.0 SD. A value of *w* = 100$/\bar {m}$ was therefore used in all simulations. For the upper limb torque generators, *m*_*i*_ was set equal to $\bar {m}$. The same value of *w* = 100$/\bar {m}$ was used in all simulations for all subjects, both pre- and post-limb loss; the value of *w* was not further adjusted to necessarily achieve a realistic metabolic cost in every simulation, nor to minimize the change in metabolic cost with limb loss, nor any particular pre-determined result. Keeping the same value of *w* in both the pre- and post-limb loss simulations aligns with the goal of this study in isolating the effect of limb loss.

The optimal control problems were converted to nonlinear programming problems and solved using a direct collocation method in Moco 0.4.0 software (Dembia et al., 2020). Briefly, the model’s state and control variables were discretized on a temporal grid of 101 nodal values spaced evenly over the stride duration. Simulation results in model development were invariant to finer grids tested up to 401 nodes per stride. These nodal values were optimized to minimize Eq. (3), subject to constraints of the skeletal equations of motion, muscle activation and contractile dynamics, and task constraints of kinematic periodicity and average speed. This method solves the same optimal control problem as the traditional forward dynamics approach in biomechanics (*e.g.*, Neptune, Kautz & Zajac, 2001) but avoids integrating the model’s state equations forward in time, which allows for high-resolution muscle excitations and relatively fast computational speeds (Van den Bogert, Blana & Heinrich, 2011). The constraint tolerance for convergence of the optimizations was 10^−4^.

#### Validation simulations

**Figure 3:**
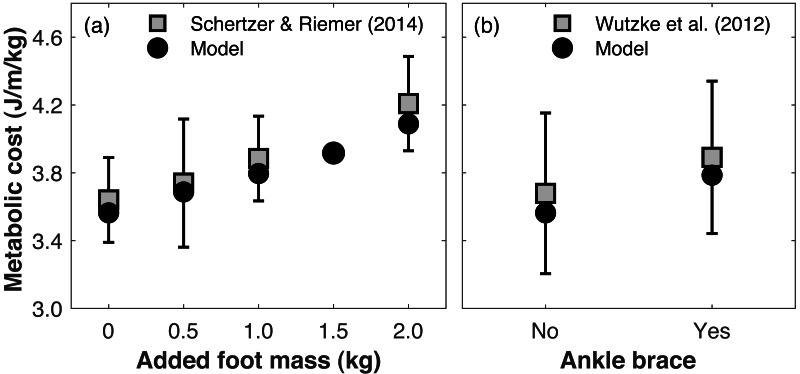
Gauging validity of model’s metabolic cost predictions *vs.* human experimental results. Metabolic costs of simulations with (A) added foot mass or (B) an ankle brace, compared to human experimental data that tested similar conditions. Error bars are one standard deviation around the experimental mean.

Directly validating the model’s ability to predict changes in metabolic cost with limb loss is not possible since there are no longitudinal data on changes in metabolic cost pre- *vs.* post-limb loss. To gauge the model’s validity for predicting changes in metabolic cost, we compared its predictions to literature reports on how metabolic cost changes when altering lower leg mechanics. Schertzer & Riemer (2014) added 0.5, 1.0, and 2.0 kg mass to each foot (weights around the ankles) and reported average respective increases in metabolic cost of +2.9, 6.7, and 16.0%. We added the same masses to our pre-limb loss model and simulation approach above, using the subject whose metabolic cost was closest to Schertzer & Riemer’s (2014) mean cost with no added mass. The masses were added to the model by increasing the mass of the calcaneus segment. The cost function and simulation procedure was the same as described in ‘Overview’. The simulated metabolic costs at all three added mass levels (+3.5, 6.5, 14.7%) were within one standard deviation of the mean from the experimental data (Fig. 3A). We also evaluated the model’s prediction of metabolic cost with ankle bracing. Wutzke, Sawicki & Lewek (2012) used a unilateral ankle brace that reduced ankle range of motion by 13° and reported an average increase in metabolic cost of 5.8%, when assuming the same resting metabolic rate as the model. We simulated an ankle brace by adding a linear torsional spring to the model’s right ankle and generating a walking simulation, again using the cost function and simulation procedure described in ‘Overview’. With a spring stiffness of 1,000 Nm/rad, the ankle range of motion was reduced by 16°, and the metabolic cost increased by 6.2% (Fig. 3B).

Changing leg mass or ankle stiffness is, of course, not the same as walking with a prosthesis, but reasonably accurate responses of the model’s metabolic cost to these conditions gives some confidence in its ability to accurately predict small changes in metabolic cost for conditions that cannot easily be tested experimentally, such as pre- *vs.* post-limb loss.

#### Pre-limb loss simulations

Simulations with each subject’s pre-limb loss model described in ‘Pre-Limb Loss Model’ were performed using the approach described in ‘Overview’. The same tracking targets ${\mu }_{i} \left( t \right) $ were used in the cost function (Eq. (3)) for all subjects. Each simulation was performed with three different initial guesses generated in model development and the result with the lowest cost function score was retained. The three “initial guess” simulations were generated using the generic pre-limb loss model with different values of *w* = 10$/\bar {m}$, 100$/\bar {m}$, and 1000$/\bar {m}$ specified in Eq. (3). The initial guess for these simulations was the means of the tracking targets for the kinematic states and zeroes for the muscle states and controls. These simulations were first performed on a coarse collocation grid (11 nodes/stride) which helped with convergence when using a bad initial guess. The coarse result was then interpolated to generate an initial guess on a finer grid, and this process was repeated until a converged solution was achieved on the final 101-node grid.

For readers unfamiliar with the terms and methods of optimal control: (i) an “initial guess” provides a starting point for the optimization routine and contains all the variables to be optimized when generating a simulation, in this case the discretized values of the model’s time-varying state and control variables; (ii) multiple initial guesses are used and the result with the lowest cost function score retained because the goal of the optimization was to minimize the cost function in an absolute sense. For example, a pre-limb loss simulation that converged on a local minimum with a high metabolic cost could bias the post-limb loss simulation towards having a comparatively low metabolic cost.

#### Post-limb loss simulations

Simulations with the post-limb loss models were performed identically to the pre-limb loss simulations, with the exceptions that (i) the prosthetic limb’s ankle, subtalar, and toe joints were excluded from the tracking term in the cost function (*N*_1_ = 34 in Eq. (3)) because the tracking targets were able-bodied data, and (ii) the 13 muscles of the right ankle were removed (*N*_2_ = 79 in Eq. (3)). To approximate the patient-specific prosthesis tuning process typically done in clinical practice, the stiffness and damping parameters of the three prosthetic joints (Eq. (1)) were optimized in Moco for each subject’s post-limb loss simulation, along with the muscle excitation controls and model states, to minimize the cost function. Stiffness was bounded on 100–1,000 Nm/rad and damping on 0.01–10 Nm/(rad/s).

For all post-limb loss simulations, the same subject’s pre-limb loss simulation result was used as the initial guess. The cost function in the post-limb loss simulations used the values of ${\mu }_{i} \left( t \right) $ from the able-bodied subjects of Miller et al. (2014) as tracking targets. The post-limb loss model was therefore not explicitly trying to track gait data from individuals with limb loss. The goal of these post-limb loss simulations was to walk with minimal deviations from a typical able-bodied gait with low muscular effort, a common goal of rehabilitation after limb loss.

#### Outcome variable and statistics

The outcome variable from each simulation was the gross metabolic cost, expressed as the metabolic energy consumed per unit distance traveled, divided by the biological body mass. To be clear, the pre-limb loss metabolic cost was scaled by the pre-limb loss biological mass, and the post-limb loss metabolic cost was scaled by the post-limb loss biological mass. The biological mass was 75.4 kg pre-limb loss and 72.8 ± 0.3 kg post-limb loss. Scaling by biological body mass (excluding the prosthesis mass in the post-limb loss case) was done to avoid favoring the hypothesis of similar metabolic costs pre- *vs.* post-limb loss, which would have been favored by scaling the post-limb loss metabolic costs by the larger total body mass that includes the prosthesis.

The effect of limb loss on metabolic cost was tested first with a standard Welch’s *t*-test to determine if the null hypothesis of no change could be rejected. Equivalence of the pre- *vs.* post-limb loss metabolic costs was then tested using two one-sided tests (Lakens, Schell & Isager, 2018):

\begin{eqnarray*}d\leq -{d}_{min}\nonumber\\\displaystyle d\geq {d}_{min} \end{eqnarray*}

where *d* is the effect size in the data for the post-limb loss change in metabolic cost, and *d*_*min*_ is the minimum effect size of interest, defined as a 2% change earlier. When both of these hypotheses can be rejected, the change in metabolic cost lies statistically within the equivalence bounds of −*d*_*min*_ ≤ *d* ≤ *d*_*min*_, suggesting the pre- *vs.* post-limb loss metabolic costs are practically equivalent.

## Results

### Gait mechanics

The pre-limb loss model tracked the mean experimental 3-D kinematics and GRFs with an average deviation of 0.51 SD. The post-limb loss model had an average deviation of 0.65 SD, excepting the joint angles of the prosthetic limb which were not tracked. Small non-zero GRFs appear in the “swing” phase from the foot briefly clipping the ground as the leg is swung forward.

**Figure 4:**
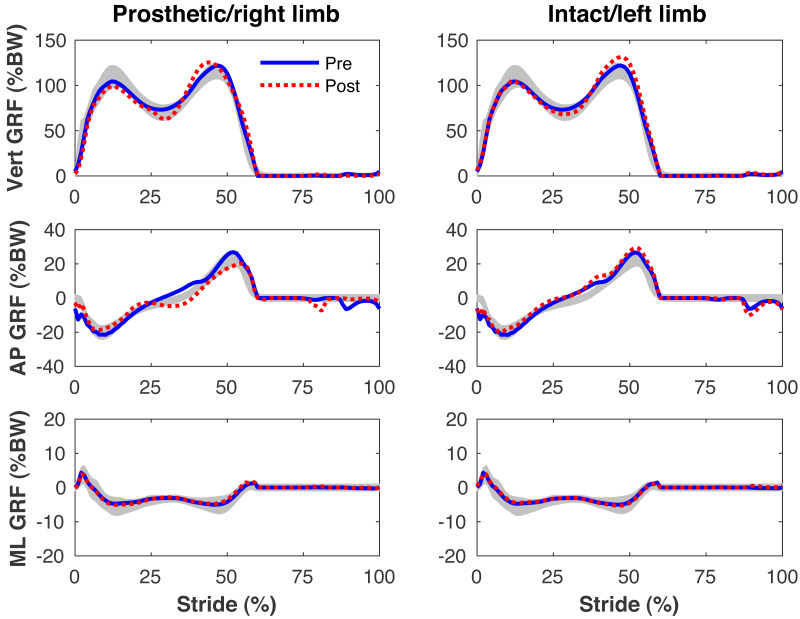
Simulated ground reaction forces. Ground reaction forces (GRF) in the vertical, anterior-posterior (AP), and medial-lateral (ML) directions during the stride cycle for the pre-limb loss (solid lines) and post-limb loss (broken lines) simulations. Data begin and end at heel-strike of the leg indicated in the column headings. Shaded areas are ± one standard deviation around the mean of instrumented gait analysis measurements from humans walking at the same average speed (Miller et al., 2014).

**Figure 5:**
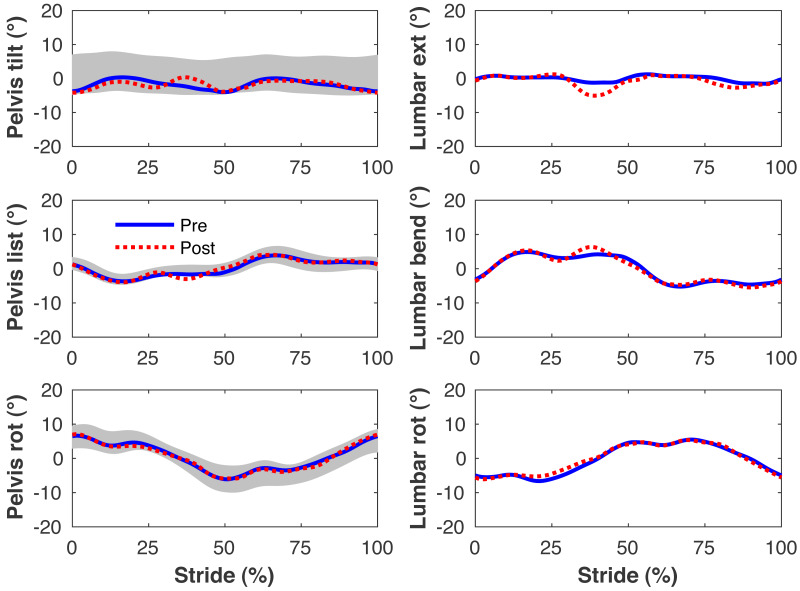
Simulated lumbo-pelvic kinematics. Pelvis and lumbar joint angles during the stride cycle for the pre-limb loss (solid lines) and post-limb loss (broken lines) simulations. Data begin and end at heel-strike of the right leg, the prosthetic leg post-limb loss. Shaded areas are ± one standard deviation around the mean of instrumented gait analysis measurements from humans walking at the same average speed (Miller et al., 2014).

**Figure 6:**
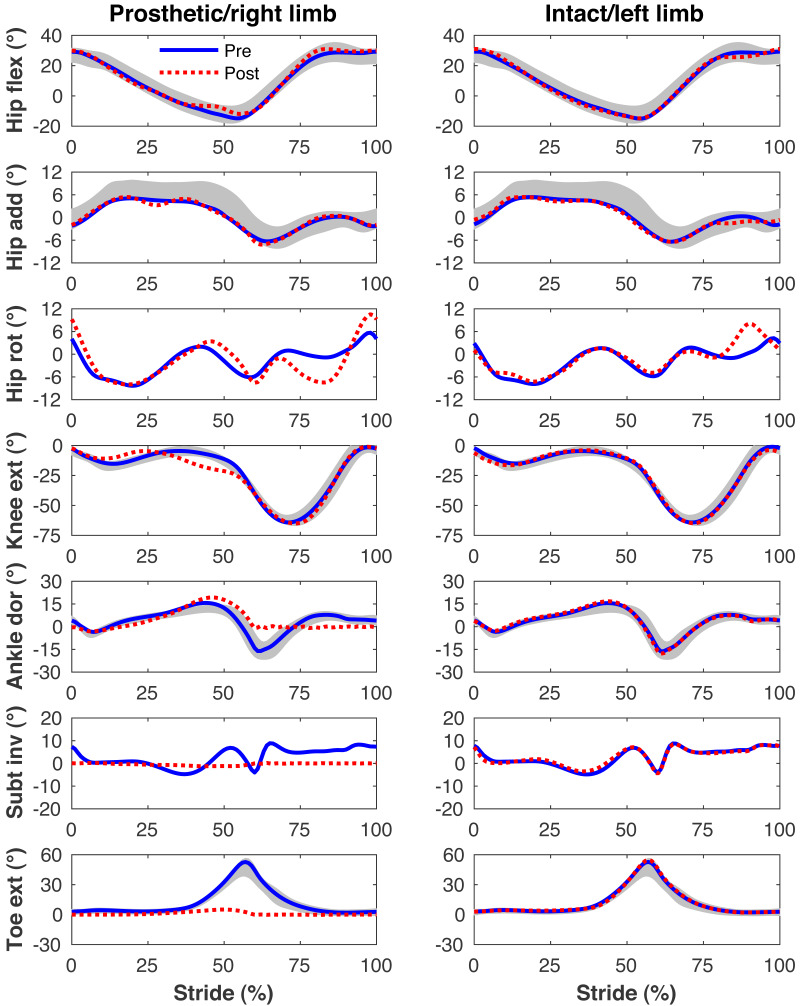
Simulated lower limb kinematics. Lower limb joint angles during the stride cycle for the pre-limb loss (solid lines) and post-limb loss (broken lines) simulations. Data begin and end at heel-strike of the leg indicated in the column headings. Shaded areas are ± one standard deviation around the mean of instrumented gait analysis measurements from humans walking at the same average speed (Miller et al., 2014).

**Figure 7:**
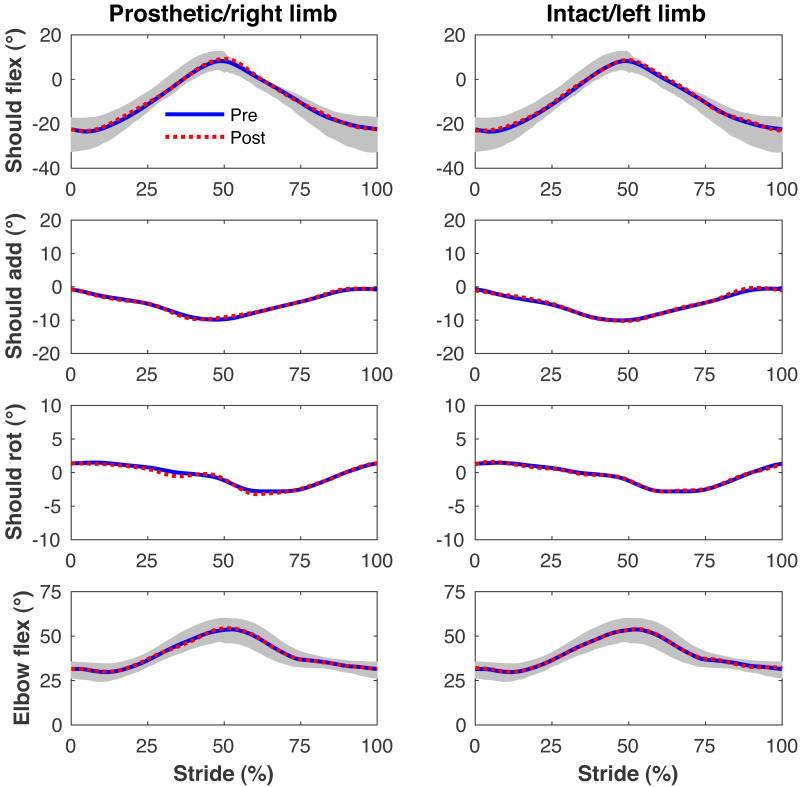
Simulated upper limb kinematics. Upper limb joint angles during the stride cycle for the pre-limb loss (solid lines) and post-limb loss (broken lines) simulations. Data begin and end at heel-strike of the leg indicated in the column headings. Shaded areas are ± one standard deviation around the mean of instrumented gait analysis measurements from humans walking at the same average speed (Miller et al., 2014).

Figures 4–7 present the time series of pre- and post-limb loss GRFs, lumbopelvic kinematics, lower limb kinematics, and upper limb kinematics, respectively, averaged over subjects. Compared to pre-limb loss, the peak anterior GRF on the prosthetic limb was reduced after limb-loss (26.7 ± 0.5 *vs.* 21.2 ± 0.6%bodyweight, *p* < 0.001; Fig. 4), and the second peaks of the vertical GRF became bilaterally asymmetric, with on average 26% less vertical force under the prosthetic limb *vs.* the intact limb (106 ± 7 *vs.* 132 ±1%bodyweight, *p* < 0.001; Fig. 4). Similar differences in GRFs have been reported in high-functioning individuals with transtibial limb loss *vs.* controls (Sanderson & Martin, 1997). Although kinematics were not evaluated statistically, these changes in GRFs were accompanied by numerous small/subtle changes in the lumbopelvic and lower limb kinematics post-limb loss of ∼5° or less, but few larger changes (Figs. 5 and 6). Limb loss did not affect upper limb kinematics (Fig. 7).

### Metabolic cost

**Figure 8:**
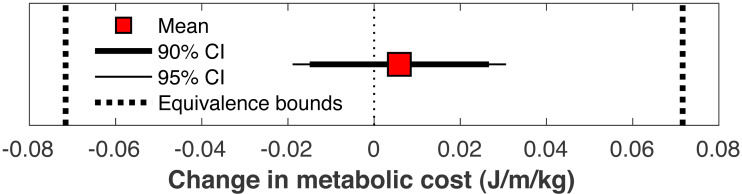
Mean change in metabolic cost with confidence intervals for TOST procedure. Mean change in metabolic cost from pre- to post-limb loss, with 90% and 95% confidence intervals. The change is was not significantly different from zero since the 95% confidence interval included zero, and was significantly less than the minimum effect of interest since the 90% confidence interval did not intercept either equivalence bound.

**Figure 9:**
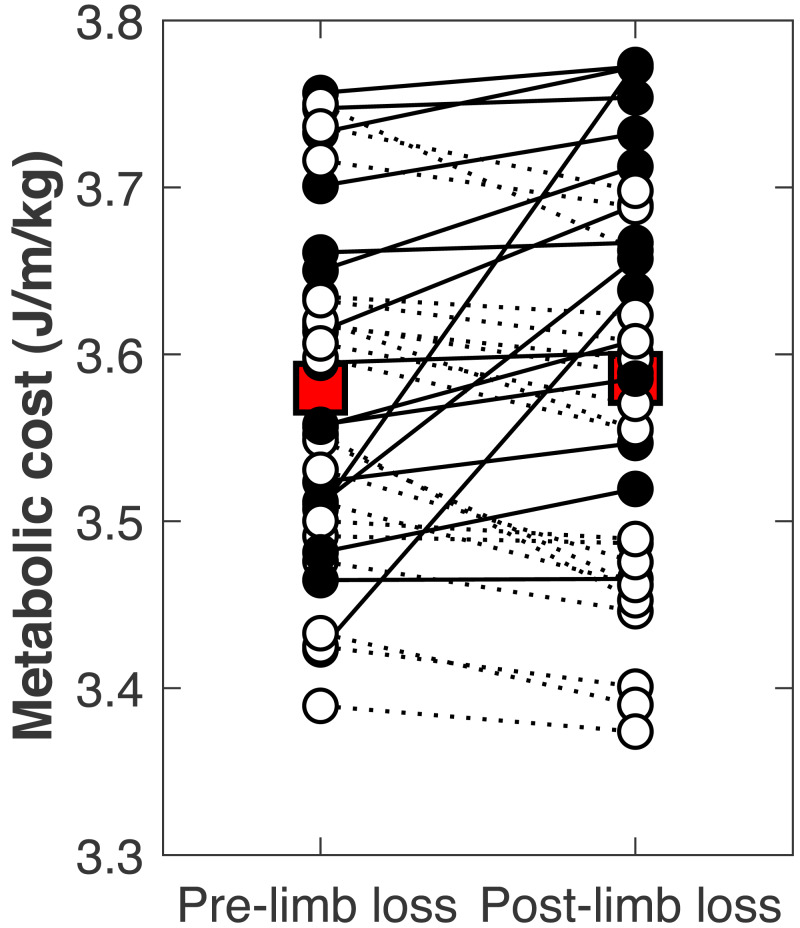
Subject-specific metabolic costs. Metabolic costs for each of the 36 subjects, pre- and post-limb loss. Symbol pairs with closed circles and solid lines had an increased cost post-limb loss. Symbol pairs with open circles and broken lines had a decreased cost post-limb loss. The larger square symbols are mean costs for each condition.

There was no significant change in the metabolic cost of walking pre-limb loss *vs.* post-limb loss (3.58 ± 0.10 J/m/kg pre-limb loss *vs.* 3.59 ± 0.12 J/m/kg post-limb loss, *p* = 0.65). The change in cost on average (+0.006 J/m/kg) fell within the equivalence bounds of   ±  0.068 J/m/kg at the 95% confidence level (*p* < 0.001; Fig. 8). Twenty of the 36 subjects (56%) decreased metabolic cost post-limb loss. The changes in metabolic cost were generally small: of the 36 subjects, only seven (19%) had a change outside the equivalence bounds of   ±  0.068 J/m/kg (Fig. 9).

**Figure 10:**
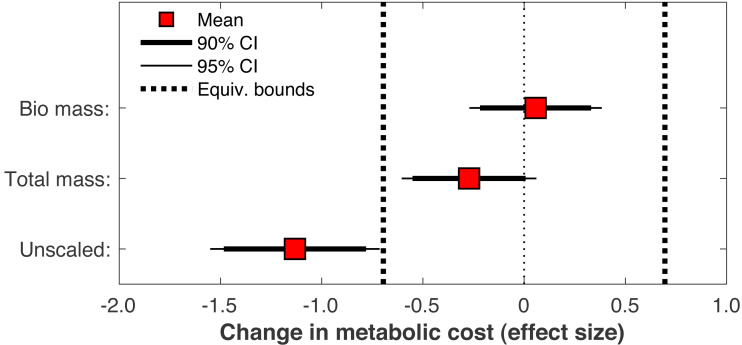
Effect of body mass scaling on change in metabolic cost. Effect sizes for mean change in metabolic cost from pre- to post-limb loss if the post-limb loss metabolic cost was scaled by the biological body mass (excluding the prosthesis), by the total body mass (including the prosthesis), or unscaled by any mass. A change is significantly different from zero if the 95% confidence interval does not cross zero, and is significantly less than the minimum effect of interest is the 90% confidence interval does not intercept either equivalence bound.

When the post-limb loss metabolic cost was scaled by the total system mass (including the prosthesis mass) instead of the biological body mass, the change in metabolic cost post-limb loss averaged −0.028 J/m/kg. This change was significant (*p* = 0.030) but still fell within the equivalence bounds (Fig. 10). When no mass scaling was performed, metabolic cost decreased post-limb loss (average −8.8 J/m). This change was significant (*p* < 0.001) and also outside the equivalence bounds (Fig. 10).

**Figure 11:**
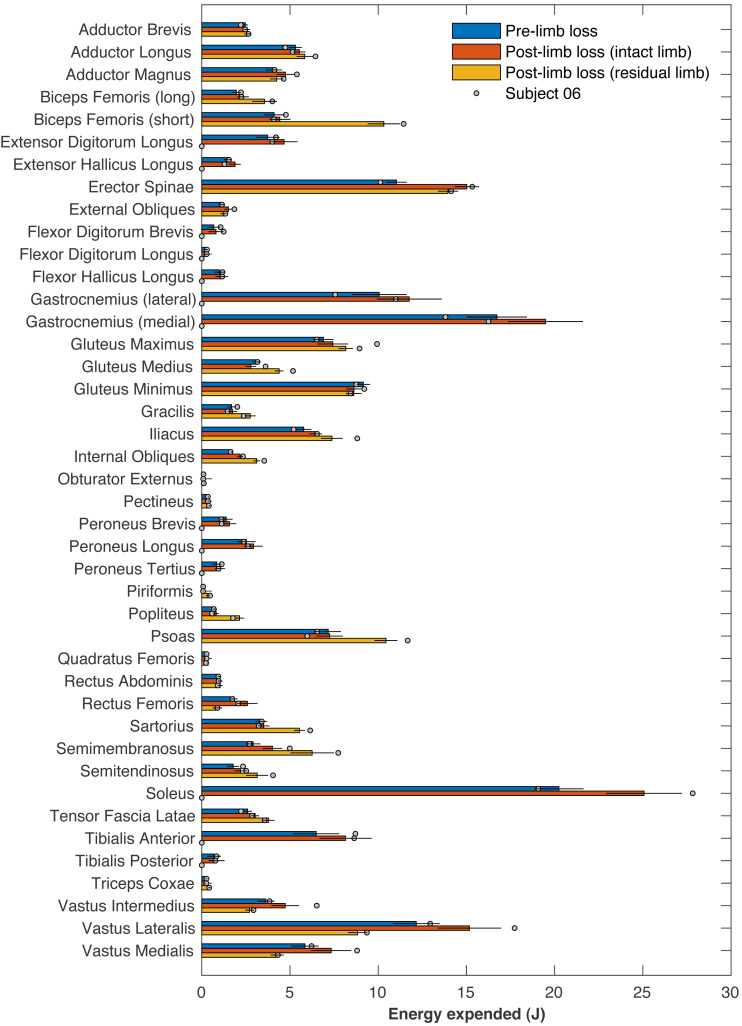
Energy expended by individual muscles. Metabolic energy expended during the walking stride by each of the model’s muscles for the pre-limb loss condition (average of both left and right legs), the post-limb loss intact limb, and the post-limb loss residual limb. Horizontal bar lengths are means over 36 subjects and error bars are standard deviations. Circles are values for Subject #06, who had the largest increase in metabolic cost post-limb loss.

Table 1 presents the pre- and post-limb loss metabolic costs and their work- and heat-related components. The right ankle muscles on average accounted for 65 J of metabolic energy, or 17% of the pre-limb loss metabolic cost. These muscles were absent and consumed no energy post-limb loss, but these savings were coupled with increases in energy expenditure by most other muscles post-limb loss (Fig. 11): the energy expended by the remaining 71 muscles post-limb loss exceeded their pre-limb loss energy expenditure by 52 J on average. The post-limb loss model still expended less total metabolic energy than the pre-limb loss model (378 J *vs.* 391 J on average), but when divided by the slightly lower biological body mass post-limb loss (72.8 kg *vs.* 75.4 kg), the metabolic cost of transporting a unit biological mass by a unit distance did not differ pre-limb loss *vs.* post-limb loss.

**Table 1:** Breakdown of energy expenditure. Total metabolic cost per kg biological body mass, and its components. Values of mean ± standard deviation for the pre- and post-limb loss simulations. The net work and heat sum to the metabolic cost. The concentric and eccentric work sum to the net work. The activation, shortening, and basal heat sum to the net heat. “Whole body” includes all muscles in the model. “Right ankle” includes only the muscles spanning the right ankle in the pre-limb loss model, which were absent in the post-limb loss model. Work-related quantities are the work of muscle fibers, except for the prosthesis, which is the mechanical energy stored/released by the prosthesis. This prosthesis energy does not directly factor into the metabolic cost so is not included under the whole-body energy components. *: The basal heat here is the non-muscular basal heat rate. The basal heat of muscles (1.0 W per kg muscle mass) was included under the muscle activation and shortening heat. The biological body masses were 75.4 kg pre-limb loss and 72.8 ± 0.3 kg post-limb loss. The bottom eight rows present the Joules of energy consumed, unscaled by body mass.

	**Pre-limb loss**		**Post-limb loss**		
**Quantity (J/m/kg)**	**Whole body**	**Right ankle**	**Whole body**	**Prosthesis**	
METABOLIC COST:	3.58 ± 0.10	0.59 ± 0.02	3.59 ± 0.12	–	
NET WORK:	0.55 ± 0.04	0.28 ± 0.01	0.61 ± 0.03	−0.01 ± 0.00	
Concentric:	1.36 ± 0.04	0.39 ± 0.01	1.28 ± 0.05	0.36 ± 0.02	
Eccentric:	−0.81 ± 0.03	−0.11 ± 0.01	−0.67 ± 0.03	−0.37 ± 0.02	
NET HEAT:	3.03 ± 0.08	0.31 ± 0.01	2.98 ± 0.09	–	
Activation:	2.14 ± 0.09	0.24 ± 0.01	2.13 ± 0.09	–	
Shortening:	0.40 ± 0.01	0.07 ± 0.01	0.33 ± 0.02	–	
*Basal:	0.49 ± 0.01	–	0.51 ± 0.01	–	
**Quantity (J)**				
METABOLIC COST:	391 ± 11	65 ± 2	378 ± 12	–	
NET WORK:	60 ± 5	31 ± 2	64 ± 3	−0.5 ± 0.2	
Concentric:	149 ± 5	42 ± 2	135 ± 5	26.6 ± 1.6	
Eccentric:	−89 ± 3	−12 ± 1	−71 ± 3	−27.1 ± 1.6	
NET HEAT:	331 ± 9	34 ± 1	314 ± 10	–	
Activation:	234 ± 10	26 ± 1	225 ± 10	–	
Shortening:	44 ± 2	8 ± 1	35 ± 2	–	
*Basal:	54 ± 1	–	54 ± 1	–	

### Prosthesis parameters

The optimized prosthesis stiffness parameters (*k* in Eq. (1)) averaged 426  ± 51, 540 ± 63, and 306 ± 90 Nm/rad for the ankle, subtalar, and toe joints, respectively. The optimized damping parameters (*b* in Eq. (1)) averaged 0.06  ± 0.06, 1.27 ± 0.52, and 0.73 ± 0.91 Nm/(rad/s) for the ankle, subtalar, and toe joints, respectively. Owing to the low damping, the prosthesis had high-efficiency energy return, storing an average of 27.1 J of mechanical strain energy and returning an average of 26.6 J (Table 1).

Figure 11 includes the energy expended by each muscle for Subject 06, who had the largest absolute increase in metabolic cost (+0.27 J/m/kg, or about +8%). No particular muscle or small group of muscles explained this subject’s increase in cost. This subject did not have outlier values for their optimized prosthesis parameters: ankle, subtalar, and toe stiffness parameters were 409, 544, and 233 Nm/rad, and the associated damping parameters were 0.20, 1.20, and 0.35 Nm/(rad/s).

## Discussion

The purpose of this study was to determine the change in metabolic cost when walking with a transtibial prosthesis in three-dimensional optimal control simulations. With a computer modeling approach, this study performed a pre- *vs.* post-limb loss comparison that is difficult to make in experiments on human subjects. Our hypothesis of similar pre- and post-limb loss metabolic costs using this three-dimensional model was supported in walking simulations. While we did not test for the effect of muscle strength as we did previously with 2-D models of limb loss (Russell Esposito & Miller, 2018), the post-limb loss model was identical to the pre-limb loss model in all parameter values including muscle strength, body mass, and mass distribution. The only difference between the pre- and post-limb loss models was the loss of the ankle muscles on the amputated side, and the replacement of the biological foot/ankle complex with a prosthetic one. This result joins a growing body of evidence suggesting that below-knee limb loss *per se* does not directly or inevitably cause the relatively high metabolic cost of walking seen in the general limb loss population (Jeans, Browne & Karol, 2011; Russell Esposito et al., 2014; Russell Esposito & Miller, 2018). An increase in metabolic cost may instead be due to changes that tend to accompany limb loss such as loss of muscle strength or other fitness- and mobility-related factors such as balance or endurance (Hewson, Dent & Sawers, 2020).

This study was hypothesis-driven and addressed a research question with a yes/no answer, “Are metabolic costs equivalent pre- *vs.* post-limb loss?” The suggested mechanism for this result is that limb loss removes the metabolic cost of the lost ankle muscles but increases metabolic cost of other muscles to compensate for the lost function (Table 1; Fig. 11), with the caveat that these compensations do not necessarily need to exceed the pre-limb loss metabolic cost. The natural follow-up questions of why metabolic cost did not change on average (Fig. 8) or why some of the 36 “subjects” had different magnitudes and directions of change (Fig. 9) are interesting but are difficult to address with confidence from the present data. For example, the subject with the largest increase in metabolic cost post-limb loss did not have an obvious muscle or muscle group that appeared to explain this increase (Fig. 11), and their model parameters, prosthesis parameters, and tracking accuracy in either simulation were not unusual. Isolating the cause of this subject-specific response would require additional simulations to discover why metabolic cost sometimes increased or decreased by relatively large amounts for some of the subjects, and to determine if this effect can be induced in a predictable way.

The model’s tracking accuracy of human gait data (Figs. 4–7) was similar to other studies that have used this simulation method with simpler models (Van den Bogert, Blana & Heinrich, 2011; Nguyen et al., 2020) and is generally better than what can be achieved with the traditional “single shooting” approach to optimal control simulations in biomechanics (*e.g.*, Miller, Esterson & Shim, 2015), owing to the ability to use high-resolution muscle excitations that more closely resemble human neuromuscular control. The differences in ground reaction forces pre- *vs.* post-limb loss were similar to those reported in high-functioning individuals with transtibial limb loss *vs.* controls (Sanderson & Martin, 1997) but the post-limb loss simulations did not exhibit the magnitude of kinematic deviations typically seen in high-functioning individuals with transtibial limb loss (Rábago & Wilken, 2016), most of which could be reasonably expected to influence metabolic cost. As noted earlier, non-zero GRF appeared in the “swing” phase from the foot briefly clipping the ground as the leg is swung forward. These GRF were brief and small (∼50 N), are not unusual in these types of simulations (*e.g.*, Anderson & Pandy, 2001), and appeared in both the pre- and post-limb loss simulations, and therefore did not bias the pre- *vs.* post-limb loss changes in metabolic cost. Within the limitations of the present 3-D model, it was possible to walk after unilateral limb loss with a passive prosthesis without increasing metabolic cost and without major deviations from pre-limb loss gait mechanics. This result suggests that large gait deviations, even in high-functioning individuals with unilateral transtibial limb loss, may not necessarily be due to limb loss directly. Increasing the weight on muscular effort in the cost function (parameter *w* in Eq. (3)) will generally increase gait deviations and decrease metabolic cost within a subject, but the magnitude of gait deviations did not correlate strongly with metabolic cost between subjects (*r*^2^ < 0.01 for both pre- and post-limb loss). It is difficult to comment further on possible relationships between specific gait mechanics like external work and metabolic cost (*e.g.*, Houdijk et al., 2009), as this study was not designed to determine these relationships. Relatedly, the cost function used here explicitly considered only two factors, gait deviations and muscular effort. Other unmodeled factors such as balance, comfort, and agility are also important to prosthesis users (Bell et al., 2021) and could involve trade-offs with gait deviations.

The main finding of this study of no increase in metabolic cost post-limb loss agrees with our previous conceptually similar simulation study that used a 2-D sagittal plane model (Russell Esposito & Miller, 2018). The 3-D model expends muscular effort moving and controlling itself in the non-sagittal planes, *vs.* the 2-D model where these planes did not exist and all effort was expended in the sagittal plane. The 2-D model was not simply a 3-D model that only moved in the sagittal plane, it was a model for which no other planes were defined. The present 3-D result confirms that the previous 2-D result was not due to the neglected non-sagittal mechanics and gives more confidence in the generalizability of the previous result to real human walking since it was replicated here with a more complex 3-D model. We caution though that the similarity in results between these two studies with 2-D and 3-D models should not be taken to imply that the mechanics and energetics of limb loss can necessarily be modeled realistically in 2-D as a general rule. A more conservative conclusion would be that the energy cost of frontal plane balance is similar with and without transtibial limb loss. There is limited information in the limb loss literature on the energy cost of frontal plane balance. Ijmker et al. (2014) reported that adding lateral stabilization *via* spring-cords at the waist caused small reductions in metabolic cost in individuals with transtibial limb loss (−5%) and in controls without limb loss (−3%), but an increase of 6.5% in individuals with transfemoral limb loss. However, these data do not necessarily imply that the cost/task of controlling balance in the frontal plane is unaffected by transtibial limb loss.

The muscle energy model includes the fraction of fast-twitch fibers as an input parameter, which affects the computed metabolic cost primarily through muscle activation/maintenance heat rate, which is a linear function of the fast-twitch fiber fraction (Umberger, Gerritsen & Martin, 2003). Average values from the literature were assumed for the model’s fiber-types and the same values were used for all subjects. This assumption would not be expected to have a major influence on the present results given the repeated-measures design. Our previous 2-D study used random perturbations to define fiber-types for each subject and produces the same overall finding as the present study (Russell Esposito & Miller, 2018). However, extreme cases, such as a subject with an unusually low fraction of fast-twitch fibers in the right ankle muscles and unusually high fraction elsewhere, could feasibly produce different results than the average result reported here.

Further concerning model complexity and assumptions, the research question here was whether limb loss *per se* increases the metabolic cost of walking. We therefore developed a modeling approach for testing the effect of limb loss in isolation of other factors that may affect metabolic cost, such as secondary changes in fitness, a poorly adjusted prosthesis, pain during walking, etc. While the present model is on the upper end of the complexity spectrum for models used previously in these types of simulations, the model and simulation approach required simplifying assumptions compared to real humans that should be considered when generalizing the results to real human walking. For example, as noted earlier we modeled a rigid prosthesis attachment, which was justified for the sake of simplicity by the small effect of a non-rigid attachment on the primary outcome variable (metabolic cost), but the present results would not necessarily generalize to individuals who have a loose prosthesis fit or substantial discomfort at the socket-residuum interface. For another example, we did not directly minimize metabolic cost in the cost function of the simulations because this is not currently possible in the Moco software (Dembia et al., 2020) and instead used a surrogate “muscular effort” cost. This assumption is justified by the fact that the approach predicted realistic magnitudes of metabolic cost and realistic changes in metabolic cost in comparison to human experiments (Fig. 3), but we cannot rule out the possibility that other plausible cost functions would produce different results. Lastly, the perturbations to model parameters defining different “subjects” were done only to a subset of parameters. These perturbations are intended to increase confidence in the effect of the independent variable (limb loss). Other unperturbed parameters would surely affect metabolic cost and the variance between subjects in Fig. 8, but whether they would systematically affect the change in metabolic cost with limb loss in this repeated-measures design is unclear and speculative, excluding extreme cases such a “subject” with ankle muscle and joint properties that are unusually economical for walking.

The prosthesis stiffness and damping parameters were optimized in the post-limb loss simulations to represent the clinical process of fitting/adjusting the prosthesis to the user, similar to our previous 2-D study (Russell Esposito et al., 2014). From a technical perspective, this process also ensures that the simulated gait deviations and metabolic costs are not inflated due to inadequacy in generic, unadjusted prosthesis parameters for a given subject. For all subjects the optimized stiffnesses fell within the range of stiffnesses for typical commercial prosthetic feet of ∼300–900 Nm/rad (Lehmann et al., 1993) even though bounds on these parameters allowed values outside this range, lending some confidence that the prosthesis model, although fairly simple, was reasonably realistic. The optimized damping parameters were low, as would be expected for modern prosthetic feet with efficient mechanical energy return, although some damping can theoretically be beneficial for reducing damage from high-frequency vibrations (Burnett et al., 2021). The modeling approach here could feasibly be adapted to subject-specific simulations for determining ideal prosthesis characteristics to accomplish particular patient-specific goals, similar to proposed model-based approaches on personalized gait modification and joint loading in osteoarthritis (Fregly et al., 2007).

The prosthesis model here with its low damping had an average energy return of 98% (Table 1), which is rather high compared to most conventional prosthetic feet (*e.g.*, Prince et al., 1998) and likely contributed to the post-limb loss metabolic costs, but is not necessarily unrealistic. Recent “Pro-Flex” (Össur, Reykjavík, Iceland) prosthetic feet with carbon fiber leaf springs offer considerably greater energy return than conventional feet (Childers & Takahashi, 2018), in some cases appearing to approach 100% return (Heitzmann et al., 2018), and spring-based “energy-recycling” feet with a small (0.8 W) battery can also return energy at very high rates (Zelik et al., 2011). However, prosthetic mechanical energy is not a direct component of metabolic cost and this study was not designed for causal inferences on this rather complicated relationship. For example, powering a prosthesis to act as a net generator of mechanical energy does not necessarily reduce metabolic cost (Quesada, Caputo & Collins, 2016), removing the ability of the ankle to store and return energy does not necessarily increase metabolic cost (Vanderpool, Collins & Kuo, 2008), and increasing energy return does not necessarily reduce metabolic cost (Zelik et al., 2011).

Metabolic cost data in biomechanics are often scaled by body mass for analysis. With limb loss subjects there is a choice of whether this scaling includes or excludes the mass of the prosthesis. Some studies have scaled by biological body mass excluding the prosthesis mass (Russell Esposito et al., 2014), others by total mass including the prosthesis mass (Jarvis et al., 2017), and others still by an unspecified mass (Jeans, Browne & Karol, 2011). This choice is non-trivial for research questions like the present study: for a typical transtibial prosthesis mass of 1–2 kg (Mattes, Martin & Royer, 2000), the scaling choice affects metabolic cost by ∼1–3% for body masses ranging from 70–100 kg, which can feasibly change conclusions when the minimum effect of interest is ∼2%. Here regardless of the choice of scaling, limb loss did not increase metabolic cost, but different conclusions could be reached depending on the scaling choice (Fig. 10). With no scaling, limb loss decreased metabolic cost, and the decrease was greater than the 2% minimum change of interest. With total mass scaling, inclusive of both the biological mass and the prosthesis mass, limb loss also decreased metabolic cost, but the decrease was less than the defined meaningful difference of 2%. With biological mass scaling only, limb loss did not change the metabolic cost; the average result was a small increase in cost but this increase was too small to be meaningful. We contend that to address our specific research question on whether limb loss *per se* changes metabolic cost, scaling by biological mass is the most appropriate and conservative choice since it accounts for change in biologic tissue mass that occurs with limb loss, and divides the energy consumed by a smaller mass than the total mass to avoid favoring the hypothesis of no increase in cost post-limb loss. Metabolic cost scaled by biological mass could be interpreted as a metric of efficiency, the energy expended to walk by the available biological mass that is capable of consuming energy, while scaling by total mass could be interested as a metric of economy, the energy expended to transport a unit mass. Regardless of this choice, many studies on limb loss that involved mass scaling do not report the specific mass that outcome variables were scaled by. There are certainly cases where scaling by total mass rather than biological mass is a sensible approach. We are not advocating that limb loss data should always be scaled by biological mass regardless of the research question. However, reporting the specific mass used for scaling is critical for interpreting metabolic energy expenditure data and should be mandatory.

The choice of a 2% change in metabolic cost to define the minimum “meaningful” effect of interest was based on a recent study on the reliability of modern portable metabolic units (Guidetti et al., 2018). The smallest “clinically relevant” change for walking with limb loss or for walking in general is not well defined to our knowledge, but is presumably at least as large as the minimum reliably measurable difference used here. The small value used here avoided favoring our hypothesis of no necessary increase in metabolic cost with limb loss. In addition, “small” changes in gross metabolic cost (under 5–10%) are often of interest in prosthesis research (*e.g.*, Zelik et al., 2011; Nolan, 2012).

A final limitation of note is that these results pertain to unilateral transtibial limb loss only, despite any ambiguous references in this report to general “limb loss”. We would expect similar results for simulations of lower-level limb loss, *e.g.*, mid-foot, but not for higher-level limb loss *e.g.*, above-knee or bilateral limb loss, but these expectations are speculative. Relatedly, these results do not necessarily generalize to other more demanding movements such as running or walking with substantial load carriage.

In conclusion, walking with a transtibial prosthesis after below-knee limb loss did not increase the metabolic cost in optimal control simulations with a 3-D musculoskeletal model. This finding supports our previous similar finding from a simpler 2-D model. The results further suggest that major deviations in the body’s intact joints and segments from able-bodied gait are also not an inevitable consequence of limb loss and may be due to secondary changes in fitness, or to priorities in the motor control of gait other than muscular effort. Lastly, studies on limb loss should report the mass used in scaling outcome variables.

##  Supplemental Information

10.7717/peerj.11960/supp-1 Supplemental Information 1 Raw data and processing/plotting code Each subject’s simulation output and Matlab code for generating plots presenting the data.

## Additional Information and Declarations

### Data Availability

The following information was supplied regarding data availability: Output datafiles for all pre- and post-limb loss simulations and a Matlab code for plotting the results are available in the Supplemental File. Pre- and post-limb loss OpenSim models are also included. The modeling project can be viewed at: https://simtk.org/projects/umocod.
